# Bone Mineral Density and Fracture Risk Assessment to Optimize Prosthesis Selection in Total Hip Replacement

**DOI:** 10.1155/2015/162481

**Published:** 2015-08-31

**Authors:** Þröstur Pétursson, Kyle Joseph Edmunds, Magnús Kjartan Gíslason, Benedikt Magnússon, Gígja Magnúsdóttir, Grétar Halldórsson, Halldór Jónsson, Paolo Gargiulo

**Affiliations:** ^1^Institute for Biomedical and Neural Engineering, Háskólinn í Reykjavík, Menntavegur 1, 101 Reykjavík, Iceland; ^2^Department of Rehabilitation, Landspítali, Norðurmýri, 101 Reykjavík, Iceland; ^3^Orthopaedic Clinic, Landspitali Hospital, Norðurmýri, 101 Reykjavík, Iceland; ^4^Medical Faculty, Háskóli Íslands, Sæmundargötu 2, 101 Reykjavík, Iceland; ^5^Department of Science, Landspítali University Hospital, Norðurmýri, 101 Reykjavík, Iceland

## Abstract

The variability in patient outcome and propensity for surgical complications in total hip replacement (THR) necessitates the development of a comprehensive, quantitative methodology for prescribing the optimal type of prosthetic stem: cemented or cementless. The objective of the research presented herein was to describe a novel approach to this problem as a first step towards creating a patient-specific, presurgical application for determining the optimal prosthesis procedure. Finite element analysis (FEA) and bone mineral density (BMD) calculations were performed with ten voluntary primary THR patients to estimate the status of their operative femurs before surgery. A compilation model of the press-fitting procedure was generated to define a fracture risk index (FRI) from incurred forces on the periprosthetic femoral head. Comparing these values to patient age, sex, and gender elicited a high degree of variability between patients grouped by implant procedure, reinforcing the notion that age and gender alone are poor indicators for prescribing prosthesis type. Additionally, correlating FRI and BMD measurements indicated that at least two of the ten patients may have received nonideal implants. This investigation highlights the utility of our model as a foundation for presurgical software applications to assist orthopedic surgeons with selecting THR prostheses.

## 1. Introduction

Total hip replacement (THR) is one of the most globally-utilized and successful orthopaedic procedures. THR aims to restore hip function and relieve patients of pain by replacing damaged hip joints with artificial ones. THR prostheses fixation is typically classified by two general methods: with and without using acrylic bone cement. In cemented THR, the stem is fixed with bone cement, but the cementless procedure relies on extant tensile properties of the femoral head as the method of securing prosthesis stability. The use of cementless fixation is the preferable procedure for two main reasons: firstly, eventually all THR prostheses will fail and require revision or replacement surgery, a phenomenon that usually occurs more than 10 years after operation. Although loosening of the stem is the typical cause for this periprosthetic failure, cementless stems mechanotransductively induce bone ingrowth and perform better in the long term, compared to cemented prostheses that typically fail earlier due to cement degradation [1–4]. Secondly, when revision surgery does eventually occur, cementless stems are more easily removed and result in fewer surgical complications, which mainly arise from residual cemented bone being removed by cement extraction from the femoral canal [5–8]. Furthermore, when the cemented stem starts to show signs of loosening, migration of the stem within the canal leads to smoothening of the endosteal surface which can make subsequent fixation very difficult [9]. Despite the preferred avoidance of cementation, orthopedic surgeons must often prescribe cemented THR to patients whose femurs may not be capable of withstanding the tensile forces in press-fitting insertion of a cementless stem; if the periprosthetic bone is not strong enough, in-surgery femoral fracture can occur, introducing a very serious complication.

The hip is a load bearing joint, constantly subjected to high loads which lead to the gradual degradation of articular surfaces. Over time, this degradation (arthrosis) can cause functional impairment and pain. With increasing life expectancies in many populations worldwide, THR rates have increased considerably over the last few decades and are projected to continue to increase in the future [10].

Currently, there is no reliable method for quantitatively choosing between the cemented and cementless implant procedures in THR, despite the prevalence of periprosthetic fracture and unloading events in many THR patients. In most cases, it is still the respective opinion of the physicians involved that dictates this decision, an opinion typically founded upon both the surgeon's own experiences and qualitative generalizations based on possible indicators of bone quality (gender, age, and qualitative assessment of CT images). In general, there is a great need for a robust, quantitative method to securely choose the appropriate implant on an individual patient basis.

**Figure 1:**
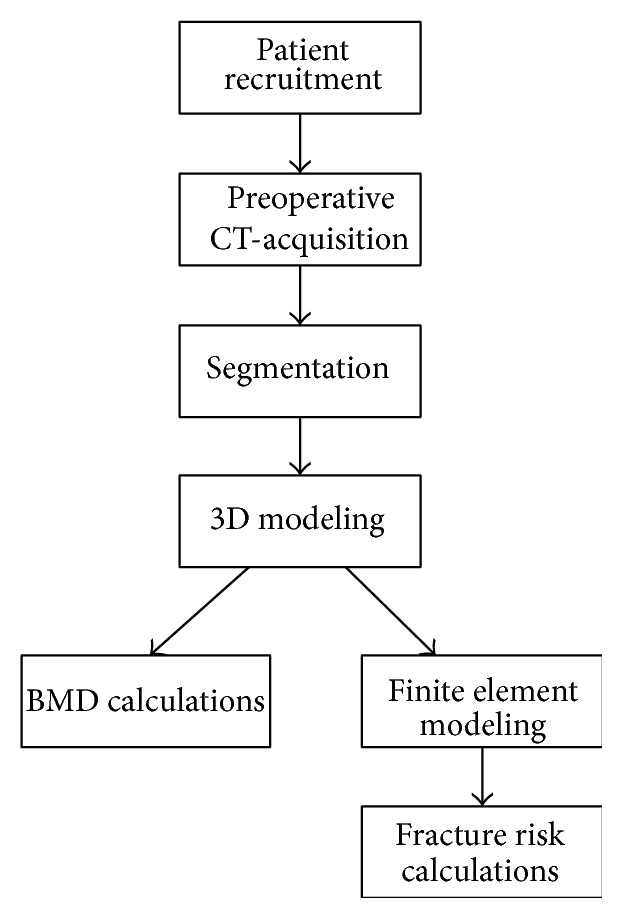
Study workflow.

Many finite element studies on total hip replacement focus on the boundaries between the femoral bone, the cement, and the stem as well as the remodeling procedures of the bone due to changes in localized stresses within the tissue. The focus of this study was to evaluate the preoperative status of the bone by introducing a novel method for fracture risk index (FRI) computation and comparing this assessment to CT-based measurements of patient bone mineral density (BMD). The reported model relies on computer tomography (CT) images to build 3D models of the femur and perform localized finite element analyses (FEA). Likewise, femoral BMD is calculated on the proximal femur as an additional metric for quantitative assessment. This method provides a patient-specific estimation of the risk of preoperative fracture, which may be utilized by an orthopedic surgeon as a tool for THR surgical planning. The work flow for this study can be seen in Figure 1. Optimizing the preoperative planning can increase the overall success of THR surgeries and have a profoundly beneficial impact on both patient mobility and lessening the economic burden of revision surgeries within many healthcare systems.

## 2. Material and Methods

### 2.1. Patient Recruitment

Ten patients were voluntarily enrolled in the study (eight females and two males). Of these patients, five patients received cementless implants, while five received cemented implants. The implant type was decided according to the evaluation of the surgeons, qualitatively based on age, sex, and general physical condition, as typically assessed before surgery. The average age at the time of operation was 61.4 ± 10.1 years for all patients. Ages averaged 63.5 ± 17.7 years for males and 54.5 ± 18.9 years for females, and when grouped according to implant procedure, average ages were 55.0 ± 9.5 years for cementless and 67.8 ± 5.8 years for cemented. Patients with total knee implants, previous hip implants, or those who received implants during this research period were excluded from the study.

### 2.2. CT Acquisition

**Figure 2:**
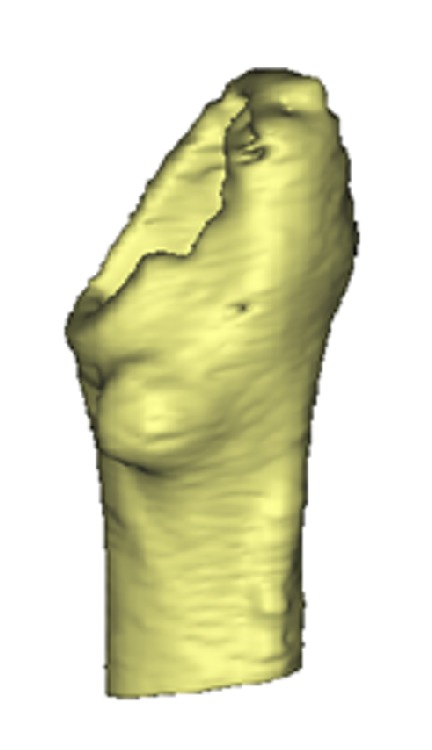
CT scanning protocol range on the femoral head.

All participants in the project were scanned with a 64 Philips Brilliance spiral-CT machine. Scanning occurred at three time points: immediately before surgery and 24 hours and 52 weeks after surgery. For the purpose of this study, only preoperative and 24-hour postoperative data were used. The scanning region extended from the iliac crest to the middle of the femur (Figure 2). The image protocol included slice thicknesses of 1 mm, with slice increments of 0.5 mm and the tube intensity set to 120 keV.

Prior to the study, the CT scanner was calibrated using a Quasar phantom to acquire the relationship between HU and BMD, resulting in the relationship given by

(1) $$\begin{array}{c} \text{BMD}\;\left[\;\frac{\text{g}}{{\text{cm}}^{3}}\;\right]=\left(\;0.00036\;\right)\text{HU}+0.56736. \end{array}$$

Linear regression analysis of this calibration resulted in a correlation coefficient *R* ^2^ ~ 0.99.

### 2.3. Segmentation and Finite Element Modeling

In order to assemble the 3D models of each patient's femur for FEA analysis, each patient's preoperative CT scan was imported into MIMICS Software (Materialise, Belgium) where femoral contour segmentation was carried out. A solid 3D model was calculated based on these contours. Next, the femoral head was virtually cut, similarly to a typical THR surgical procedure, using Boolean operators on the 3D model. Additionally, a virtual distal cut was performed orthogonal to the femur's long axis. The final model can be seen in Figure 2.

**Figure 3:**
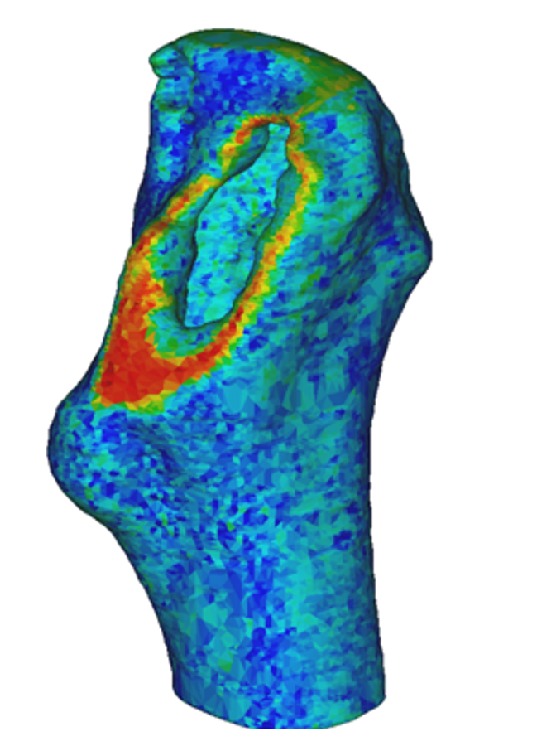
A finite element model of the femur consisting of more than 100,000 elements. The elements are given material properties, namely, Poisson's ratio and Young's modulus. Red colours indicate higher density bone.

Using the finite element module of MIMICS, known as 3-Matic, the model was divided into quadnode tetrahedral elements. Each model consisted of 130,000 to 170,000 of these elements, with overall element densities of around two elements per mm^3^. Young's modulus and Poisson's ratio were then assigned to each element. Fifty different values of Young's modulus were assigned to the elements of each model, while the Poisson's ratio was considered a constant value of 0.33. Furthermore, these elements were considered to be isotropic. The aforementioned calibration equation was used to convert HU to BMD and (2) was used to convert BMD to Young's modulus [11]:

(2) $$\begin{array}{c} E=10500\cdot {\rho_{\text{ash}}}^{2.29}, \end{array}$$

where *ρ*_ash_ is the bone mineral density obtained from (1). This formula was used to represent both trabecular and cortical bones. In Figure 3, a FE model of a respective femur can be seen, following the addition of material properties.

### 2.4. Fracture Risk Index Computation

**Figure 4:**
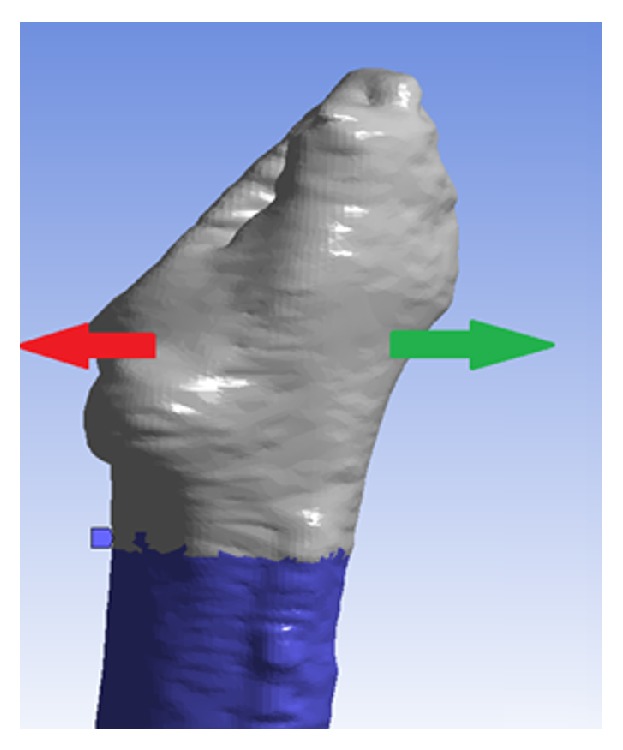
The 9.25 kN cementless prosthesis forces applied on the model where the highest stress can be expected during the press-fitting of the tapered stem.

In order to compute the FRI of each femur, each FEA model, complete with requisite material properties, was imported into Ansys Mechanical APDL v.14.0 (©ANSYS, Inc.). There, a static structural simulation and analysis were performed on the model. The objective of this simulation was to simulate the forces introduced on the femur during the press-fitting surgery in cementless THR. In this procedure, when the stem is pushed into the medullary canal, the highest tensile stresses can be expected to arise at the medial and lateral sides of the periprosthetic end of the femur. This is due to the fact that the flare of the stem is the steepest at the top. Therefore, as boundary conditions, two equal but opposite forces were applied in these areas. In a study by Sakai et al. the average measured hammering force for uncemented prosthesis was estimated to be 9.25 kN [12]. Since the forces in cemented prosthesis are considerably lower, our model utilized this force value as a worst-case-scenario to discern whether any of the ten patients could have withstood the cementless method (Figure 4).

To determine the FRI for each model, the stress value of every element was compared to its calculated ultimate tensile strength (UTS). The ultimate tensile strength was calculated with a relationship given by Bessho et al. ((3) and (4)) [13]:

(3) UTS137·BMD1.88for  BMD<0.317,

(4) UTS=114·BMD1.72for  BMD≥0.317.

The average stress experienced by each element was calculated by averaging the stress values at each node point. The fracture risk was calculated based on the preoperative scan, simulating for all the patients the uncemented fixation (press-fitting procedure) independent from surgeon's decision on the implant type. The fracture risk index was calculated for each element using [14]

(5) $$\begin{array}{c} \text{FRI}\left(\;\%\;\right)=\frac{\text{stress}}{\text{UTS}}\cdot 100\%. \end{array}$$

### 2.5. Bone Mineral Density Computation

**Figure 5:**
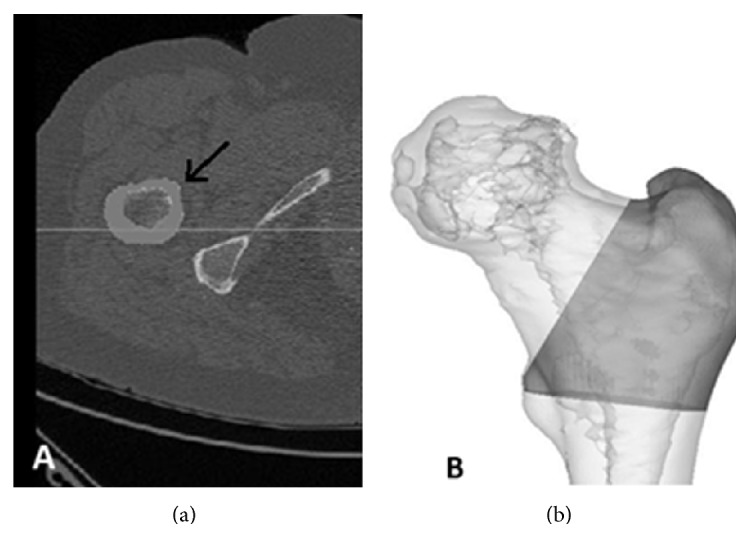
(a) Axial view of one slice of the CT-data from a patient. The arrow points to the area belonging to the mask. (b) The 3D view of the region of interest for BMD calculations.

A model of the BMD region of interest was created in MIMICS, ranging from the periprosthetic femur, without the femoral head, to the greater trochanter, with a distal axial cut through the lesser trochanter (Figure 5). The study focused on the structural aspects of the cortical bone, since the structural integrity of the cancellous bone is compromised after operation. The horizontal line in Figure 5 demonstrates the cuts made above and below the area of interest. From this region, the HU values were extracted and converted to BMD using (1).

## 3. Results

Firstly, the FRI was calculated for ten patients, applying the uncemented loading condition for all subjects independent of the surgeon's decision. From the cohort five patients received a cemented implant and five an uncemented implant. The highest stresses on the models were usually experienced in the calcar femoral on the medial side of the femur and at a similar location on the lateral side.

**Figure 6:**
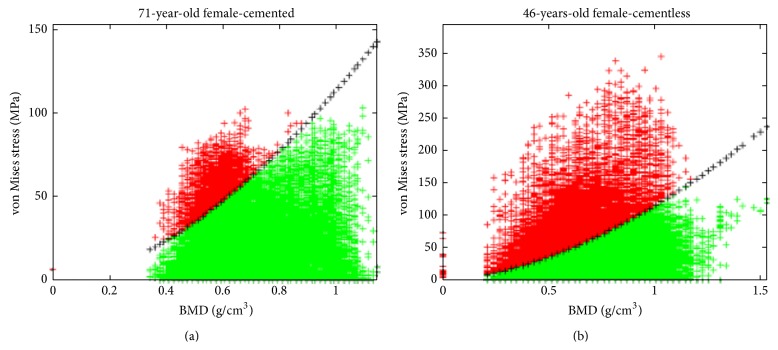
Examples of calculated FRI from (a) a 71-year-old female patient and (b) a 46-year-old female. Note that every element of the model has been plotted with von Mises stress as a function of bone mineral density. The black crossed line indicates element strength given by (3), and elements are considered failed if they surpass this line in stress. Subject B actually experienced femoral fracture due to the periprosthetic implant some days after the surgery.

In Figure 6, the von Mises stress in every element of each model is plotted against BMD. The black-crossed line represents the ultimate stress as a function of BMD, as stated in (3). Should the calculated stress values exceed the UTS, then the element would be considered fractured, which can be seen as a dark color in the figure.

**Figure 7:**
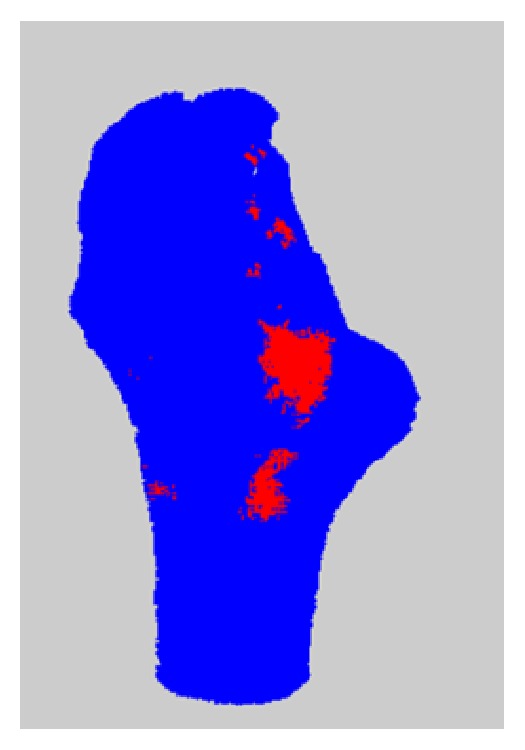
Elements plotted in their three-dimensional coordinates. Note that red elements were those that exceeded the acceptable limit for von Mises stress, indicating regions of most probable periprosthetic fraction.

Figure 6 depicts the position-independent von Mises stresses of all elements against their respective BMD values. To better visualize where the highest risk of fracture was experienced, these elements were also plotted in 3D. A typical result from the 3D plotted fracture risk index can be seen in Figure 7. The red-colored elements are those that exceeded their fracture threshold.

In Table 1, the average fracture risk is calculated for the ten patients (five with cemented THR and five with cementless), as well as the ratio of those elements that exceeded 80% of their ultimate stress value.

**Figure 8:**
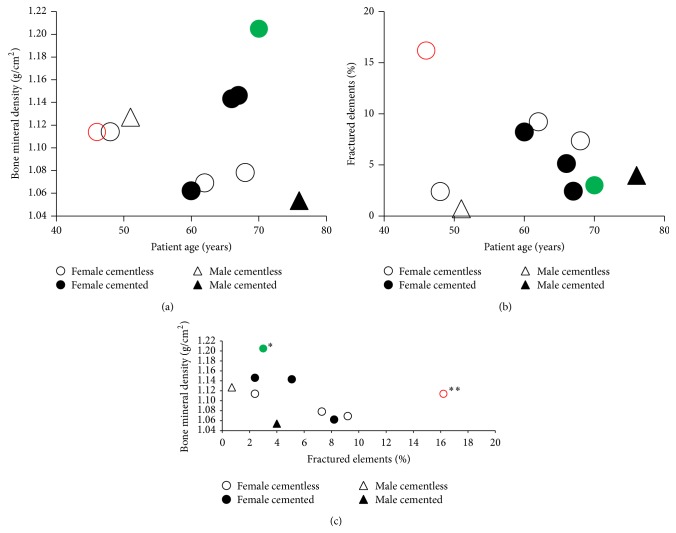
Results from FRI and BDM assessment for each patient grouped by sex and prosthesis type. (a) BMD versus patient age, (b) percent fractured elements versus patient age, and (c) BMD versus percent of fractured elements. Note that the female patients in green (^∗^) and red (^∗∗^) received nonoptimal cemented and cementless prostheses, respectively, according to our computational assessment. Furthermore, it should be noted that the red patient suffered a periprosthetic fracture during surgery, an event that could possibly have been predicted by the above results.

For the five cementless patients, the average age was 55 ± 9.5 years, the average percent of fractured elements was 7.16 ± 6.13%, and the average BMD was 1.10 ± 0.03 g/cm^2^. For the five cemented patients, the average age was 67.80 ± 5.85 years, the average percent of fractured elements was 4.54 ± 2.29%, and the average BMD was 1.122 ± 0.06 g/cm^2^. These values in relation to patient age and sex can be seen in Figure 8.

## 4. Discussion and Conclusions

The most important criterion when choosing the type of implant for patients undergoing THR is bone quality. If the bone is of good quality, the cementless implantation method generally results in fewer patient complications and generally more delayed revision surgeries, compared to cemented THR. Since bone quality tends to decline with age and is usually lower in women than men, younger and/or male patients usually receive cementless implants, while older and/or female patients receive cemented ones. Although age and gender are somewhat reliable indicators of femoral bone quality, individual differences can be vast. The reported results highlight these differences and suggest the importance of developing a novel, quantitative approach to assessing patients' femoral heads before THR surgery [15, 16].

### 4.1. Bone Mineral Density as a Potential Computational Tool in THR

The notion that patient variation in bone quality as a function of age and sex is especially evident from the BMD measurements presented herein, where several patients received cemented prostheses despite having relatively higher BMD than patients who were given the cementless type. This decision was clearly based primarily on the patients' age, as the average age of cemented patients was much higher than those of cementless patients (67.8 ± 5.8 compared to 55.0 ± 9.5, resp.). Indeed, the patient with the highest BMD measurements was not only female but also the second oldest within the assessed population. This most likely justified her receiving a cemented implant, although our model suggests that she certainly may have withstood a press-fitting with a low risk of periprosthetic fracture. Additionally, our results indicate that BMD measurements may correlate inversely, to some degree, with the percentage of fractured elements computed by our FEA simulation. This is evident, as higher BMD values indicate better bone quality and thereby a reduced chance of each element exceeding its fracture threshold. However, to determine if this relationship is indeed true, more patients would need to be assessed in a larger study. In general, the use of BMD as a metric in this investigation serves as an important first step in developing a quantitative method for computing bone quality at the moment of surgery, which may serve as a future tool for orthopedic surgeons to predict the ability for patient's femurs to handle the stresses in press-fitting a cementless THR prosthesis.

### 4.2. Fracture Risk as a Potential Computational Tool in THR

The calculated FRI for the 10 subjects additionally showed high degrees of variation between patients according to both their sex and age. Most importantly, our model shows that two of the five cementless patients had higher fracture risks than all of the five cemented patients, despite them being younger than four of the cemented patients. It is additionally critical to note that the 46-year-old female, cementless patient experienced a periprosthetic femoral fracture immediately after the surgery, which correlated with both the considerably higher fracture risk and lower BMD discerned from our computational model (Figures 8(a) and 8(b), resp.). However, with a larger population size for the reported assessment, it may be reasonably expected that a majority of younger patients, who typically receive cementless implants, would have lower risks of fracture and higher BMD than those of older patients. However, the reported results show again that patient age is not necessarily an adequate indicator of either fracture risk or bone quality; thus, implementing the computational technique that this paper introduces might serve as a better preoperative tool for orthopedic surgeons to dictate the optimal THR procedure.

### 4.3. Limitations and Future Directions

As previously mentioned, the purpose of this study was to investigate whether a novel FEA simulation of press-fitting could generate a potentially useful tool for assessing patient fracture risk indices, in combination with CT-based BMD measurement. Our results do indeed highlight the potential of this methodology and furthermore suggest the inadequacy of patient age and sex in dictating the risk of periprosthetic fracture. However, a larger patient population is requisite to rigorously show the statistical dependency of FRI on measured BMR and to define limits that correlate to additional, real cases of patient periprosthetic fracture. In addition, there are some limitations of the reported FEA and FRI computations. The greatest of these is that the simulations carried out were steady-state and did not take into consideration applied loads that are time-dependent or the prosthetic design and surface finish. The real forces induced by a surgical hammer during the surgery are high-impact and punctate forces or forces acting on the bone over a short period of time. This can instigate the development of microfissures in the periprosthetic region of the femur, leading to fractures in more extreme cases.

Overall, this study proposes a novel approach to the predictive simulation and computation of BMD and FRI during insertion of a cementless THR prosthesis. A large part of the novelty of this work lies in the fact that the bone quality was discerned at the time of surgery rather than long after surgery, incorporating both bone mineral density averages and fracture risk indices in the periprosthetic region of the femur. This real-time surgical evaluation could serve as the basis for the development of software applications that orthopedic surgeons may use to discern which prosthesis fitting procedure may be optimal for each patient on an individual basis. Such a tool could have a profound impact on THR surgical planning and serve as a model for future surgical planning software. However, the development of a patient database with which such tools may operate would require more patient data than what was acquired for the purpose of the reported work. Additionally, a more robust model would include variations in stem designs, such as material roughness, tapering degree, cross-sectional area, and coating thickness. Incorporating materials data could provide additional details regarding sheer forces applied to the bone as a result of prosthetic friction, in addition to the radial forces presented herein. Nevertheless, our results highlight the feasibility of the methodology used and can be utilized as a foundation to develop a clinical database for correlating BMD and FRI to THR patient outcomes. As an eventual software application for orthopedic surgeons, our combinatory approach of CT-based BMD measurement and FEA-based assessment of femoral fracture risk could serve as a pivotal tool in the decision making process before total hip replacement. Optimizing the preoperative planning can increase the overall success of THR surgeries and have a profoundly beneficial impact on both patient mobility and overall surgical outcome, which could significantly aid in lessening the economic burden from revision surgeries upon many healthcare systems worldwide.

## Figures and Tables

**Table 1:** FRI and BMD results from the ten cemented and cementless THR subjects.

Cementless THR procedure				Cemented THR procedure			
Age	Sex	% of elements >fracture threshold	BMD [g/cm^2^]	Age	Sex	% of elements >fracture threshold	BMD [g/cm^2^]
51	M	0.7%	1.127	76	M	4.0%	1.054
48	F	2.4%	1.114	70	F	3.0%	1.205
46	F	16.2%	1.114	66	F	5.1%	1.143
68	F	7.3%	1.078	60	F	8.2%	1.062
62	F	9.2%	1.069	67	F	2.4%	1.146
